# Molecular Effects of Glucose on *MIR503HG*-Regulated Genes in Triple-Negative Breast Cancer

**DOI:** 10.3389/bjbs.2025.15206

**Published:** 2025-12-17

**Authors:** Victoria A. Reid, Barbara Yang, Kyle Russo, Melina J. Sedano, Ramesh Choudhari, Enrique I. Ramos, Shrikanth S. Gadad

**Affiliations:** 1 Center of Emphasis in Cancer, Department of Molecular and Translational Medicine, Paul L. Foster School of Medicine, Texas Tech University Health Sciences Center El Paso, El Paso, TX, United States; 2 The University of Texas at El Paso, El Paso, TX, United States; 3 Paul L. Foster School of Medicine, Texas Tech University Health Sciences Center El Paso, El Paso, TX, United States; 4 Division of Cancer Immunology and Microbiology, Medicine and Oncology Integrated Service Unit, The University of Texas Rio Grande Valley School of Medicine, McAllen, TX, United States; 5 South Texas Center of Excellence in Cancer Research, The University of Texas Rio Grande Valley School of Medicine, McAllen, TX, United States

**Keywords:** gene expression, lncRNA, RNA-seq, triple-negative breast cancer, glucose

## Abstract

Triple-negative breast cancer (TNBC) is the most aggressive breast cancer subtype, characterized by a lack of key hormone receptors in tumor cells. As there are limited treatment options for these patients, it is crucial to understand the underlying mechanisms by which TNBC constantly evolves and evades treatments. In this regard, the pervasive nature of transcription provides a potential reservoir of transcripts, including both coding and noncoding, that TNBC leverages to sustain a proliferative advantage and support tumor growth. TNBC is affected by energy sources such as glucose, which can have a profound impact on gene expression regulation mediated by various molecules, including noncoding RNAs, at the cellular level. In this study, we demonstrate that glucose modulates the gene expression profile mediated by the microRNA-503 host gene (*MIR503HG*), which has been previously implicated in TNBC. To comprehensively characterize the impact of glucose on *MIR503HG*-regulated genes and cellular pathways, we sequenced total RNA, performed gene set enrichment analyses, and determined the relation between gene expression and patient outcomes. Analysis of gene subsets specific to various glucose environments identified clinical outcomes for breast cancer patients across different molecular subtypes. Our findings indicate that *MIR503HG* has potential as a diagnostic marker and may be useful in the clinical management of TNBC.

## Introduction

Breast cancer is the leading cancer among women worldwide. According to the World Health Organization (WHO), in 2022, 2.3 million women were diagnosed with breast cancer, and there were 685,000 deaths globally [1]. Breast cancer is heterogeneous in nature, with multiple molecular subtypes. Triple-negative breast cancer (TNBC) accounts for approximately 15%–20% of all breast carcinomas [2] and is associated with aggressive tumor phenotypes and higher recurrence rates than other subtypes [3]. TNBCs are characterized by the low levels or complete absence of key hormone receptors, particularly estrogen receptor (ER), progesterone receptor (PR), and an oncogene known as human epidermal growth factor receptor 2 (HER2), which is a target of trastuzumab [4]. The lack of these hormone receptors creates a hazardous scenario for TNBC patients, as the tumors do not respond to hormone-targeting drugs that are typically used in hormone receptor-positive cancer cases. As such, standard treatments for TNBC include surgery, radiation, and chemotherapy [5], with post-treatment recurrence rates around 50% for patients diagnosed in the earlier stages [6]. Chemotherapeutic drugs, such as anthracyclines and taxanes, are commonly used in combination to treat TNBC. Anthracyclines, like doxorubicin and epirubicin, intercalate DNA strands, leading to DNA damage and cell death [7–9]. Taxanes, such as paclitaxel and docetaxel, act by stabilizing microtubules, consequently disrupting mitosis [10]. Even though there are several chemotherapeutics available to treat TNBC, identifying novel therapeutic targets for this complex breast cancer subtype, as well as learning more about its pathology, persists as a critical problem to address [11, 12].

One of the hallmark characteristics of cancer cells is metabolic reprogramming, which enables them to thrive and rapidly proliferate in a nutrient-deficient tumor microenvironment (TME). The Warburg effect describes how cancer cells are able to survive in the TME, as they exhibit high rates of aerobic glycolysis, where the cells convert glucose to lactate regardless of the presence or absence of oxygen [13, 14]. The rewiring of their metabolic network enables the cells to meet their bioenergetic demands and promote oncogenic pathways, leading to their proliferation and metastasis [15]. Although this phenomenon is seen across a wide array of cancers, studies have shown that TNBC is highly glycolytic compared to other molecular subtypes of breast carcinomas, which might contribute to its aggressiveness and poor outcomes [16]. Recent studies have classified TNBCs into multiple subtypes based on their metabolic phenotype [17], meaning each subtype must be treated uniquely. This predicament underscores the need to develop alternative therapeutic strategies that target TNBC across diverse environmental conditions.

Several studies have reported that long non-coding RNAs (lncRNAs) play an integral role in cancer cell metabolism [18–20]. LncRNAs are non-coding RNAs that have a length of 200 nucleotides or greater and play diverse roles in the regulation of gene transcription, translation, and epigenetic modification [21]. Notably, lncRNAs are able to modulate gene regulation via both cis-acting and trans-acting mechanisms, which sets them apart from other non-coding RNAs such as small interfering RNAs (siRNAs), microRNAs (miRNAs), and PIWI-interacting RNAs (piRNAs) [22]. In recent years, numerous lncRNAs have been associated with various aspects of TNBC, ranging from its pathogenesis to therapy resistance and prognosis [23], with lncRNAs exhibiting either oncogenic or tumor-suppressive characteristics [24, 25]. The involvement of dysregulated lncRNAs in the development and progression of TNBC is an area that requires further study, due to the complexity involved in characterizing lncRNAs as well as the heterogeneous nature of TNBCs.

Although the roles of lncRNAs in TNBC are an area that has been studied, there are still many lncRNAs whose mechanisms have not been fully characterized. In this study, we investigated a specific lncRNA, microRNA-503 host gene (*MIR503HG*), whose original roles were thought to be primarily angiogenic in nature [26]. Later studies identified *MIR503HG* as being highly expressed in reproductive tissues [27], and most recently, it has been identified as an oncogene in prostate cancer [28] and as a regulator of cell differentiation and insulin production in stem cell-derived pancreatic progenitors [29]. Other studies have shown that the overexpression of *MIR503HG* can impair epithelial-to-mesenchymal transition (EMT) properties, acting as a tumor suppressor lncRNA in diseases such as ovarian cancer [30]. Furthermore, *MIR503HG* has been implicated in TNBC, in which it inhibits cell proliferation [31]. However, in the context of glucose involvement in cancer, it will be crucial to determine whether it affects *MIR503HG*-dependent gene regulation. Additionally, the role of glucose in modulating *MIR503HG*-dependent gene regulation in TNBC remains an unexplored area. Hence, the objective of this study was to identify the effects of glucose on *MIR503HG*-mediated gene expression in TNBC cells and assess its possible clinical implications. To achieve this, we treated TNBC cells engineered to overexpress *MIR503HG* with varying amounts of glucose. Subsequently, we conducted total RNA-sequencing to evaluate the impact of glucose on genes regulated by *MIR503HG*. Our RNA-seq analysis of this data has revealed that *MIR503HG*-regulated genes were modulated by glucose, altering the expression profiles of TNBC cells. Overall, our analyses have unveiled interesting biological aspects of glucose and *MIR503HG* interaction in TNBC. These findings could potentially be applied in the clinical setting to inform the development of novel therapeutic approaches.

## Materials and Methods

### Cell Lines and Cell Culture Conditions

MDA-MB-157 cells were obtained from the American Type Culture Collection (ATCC) and regularly cultured in high-glucose Dulbecco’s Modified Eagle’s Medium (DMEM) (5 g/L glucose, Sigma-Aldrich, D6429). The media was supplemented with 10% fetal bovine serum (FBS, Gibco, 26140079) and 1% penicillin/streptomycin (Gibco, 15140-122), and cells were maintained in a humidified incubator at 37 °C and 5% CO_2_. For induction of *MIR503HG* or *GFP* with doxycycline (Dox, Sigma Aldrich, D9891), the cells were treated with 250 ng/mL of Dox for the indicated times before collection. For glucose treatments, cells were incubated in low-glucose DMEM media (1 g/L glucose, Sigma-Aldrich, D6046) for 48 h, before being treated with the experimental glucose concentration (1, 5, or 15 g/L glucose) and co-treated with Dox for 4 h.

### Dox-inducible Ectopic Expression in Cell Lines

Lentiviruses were generated by transfecting HEK293T cells (ATCC, CRL-3216) with pInducer20 [32] containing *MIR503HG* or *GFP*, along with pMD2.G, psPAX2 plasmids using the Lipofectamine 3000 kit (Thermo Fisher Scientific, L3000015) according to the manufacturer’s protocol. Medium from transfected 293T cells was collected and filtered through a 0.45 µm filter to transduce MDA-MB-157 cells, and polybrene (0.5 μg/mL) was added. Stably transduced cells were selected under drug selection by Geneticin™ (G418, Gibco, 11-811-031) at 1 μg/mL and were used for Dox-induced ectopic expression of *MIR503HG* and *GFP* to perform a variety of experiments described herein.

### Total RNA Isolation, RNA-Seq Library Preparation, and Sequencing

Cells expressing inducible *MIR503HG* or *GFP* were seeded in six-well plates and treated with 250 ng/mL Dox and media with the indicated glucose concentrations for 4 h. The total RNA was isolated using EZ-10 DNAway RNA Mini-Preps Kit (Bio Basic, Canada). Agilent Technologies 4200 TapeStation was used to analyze RNA integrity of the preparation, and Nanodrop (Thermo Scientific, USA) was used to conduct RNA quantification. Only isolated RNA samples with >9 RIN (RNA Integrity Number) were utilized for RNA-seq library preparation. RNA-seq libraries for total RNA-sequencing were prepared and sequenced at Novogene Corporation (Sacramento, CA). Briefly, ribosomal RNA was removed from the total RNA and then precipitated using ethanol. After fragmentation, the cDNA was synthesized using random hexamer primers, followed by the addition of a second-strand synthesis buffer (Illumina), dNTPs, RNase H, and DNA polymerase I to initiate the second-strand synthesis. Next, terminal repair, A-tailing, and sequencing adaptor ligation steps were carried out. Finally, the double-stranded cDNA library was completed through size selection and PCR enrichment. The libraries then went through QC checks, which include Qubit 2.0, Agilent 2100, and real-time PCR. Finally, the quantified libraries were pooled according to the concentration and data amount required and fed into Illumina sequencers (NovaSeq X Plus).

### Transcriptomic Data

The transcriptomic data for this study were submitted to NCBI’s Gene Expression Omnibus and are accessible through NCBI-GEO-GSE297214. This dataset represents RNA-seq-based gene expression in the breast cancer cell line MDA-MB-157, which includes samples of G1, G5, and G15 glucose treatments for cells overexpressing *MIR503HG* or *GFP*.

### Transcriptome Assembly and Differential Gene Expression

In this study, the raw reads (fastq files) from total RNA-sequencing were uploaded to Genialis™ Expressions 3.0 [33] and processed using the General RNA-seq pipeline (featureCounts). Reads were trimmed using BBDuk (BBMap 37.90), followed by alignment to the human genome (hg38) with annotation from Ensembl release version 109 (February 2023) using STAR aligner (v2.7.10b) [34]. BAM files were then sorted and indexed using Samtools (v1.14) [35]. Read counts were quantified using featureCounts (1.6.3) [36], with reference annotation from Ensembl version 109. Normalization of expression values was calculated using rnanorm (1.3.1). The DESeq2 tool was used for count normalization and differential gene expression analyses. Filtering criteria for differentially expressed genes were set by applying a p-value (≤0.05).

### Gene Set Enrichment Analysis

Utilizing the Molecular Signatures Database (MSigDB) [37], Gene Set Enrichment Analysis (GSEA) version 4.4.0 was conducted to examine the relationship among top DEGs to specific processes and mechanisms. Top 20 gene sets (gene ontologies) with FDR q-value less than 0.05 for gene ontology (GO), KEGG, Hallmark, and CGP were generated for up- and downregulated genes. Genes were ranked by log2 Fold Change value, and the top 500 were used as the input gene set for the GSEA.

### Kaplan-Meier and Gene Expression Analyses of Breast Tumor Samples

To evaluate the prognostic value of identified differentially expressed genes specific to glucose treatment, we explored the expression of merged gene sets across breast tumor tissues. The Kaplan-Meier plots estimating overall survival (OS), distant metastasis-free survival (DMFS), and relapse-free survival (RFS) in three quantiles over 10 years were generated utilizing the Gene Expression-Based Outcome for Breast Cancer Online (GOBO) tool [38]. Genes were ranked by log2 Fold Change value, and the top 500 up- and downregulated genes were used as the input gene sets for the GOBO. Boxplots of merged gene sets across molecular breast tumor subgroups was also attained using the GOBO tool, using the same gene sets. Each box depicts the combined expression score within a subgroup, with the dotted line indicating the overall median expression across all samples.

## Results

### Glucose Regulates *MIR503HG*-Dependent Genes in a Dose-Dependent Manner

**FIGURE 1:**
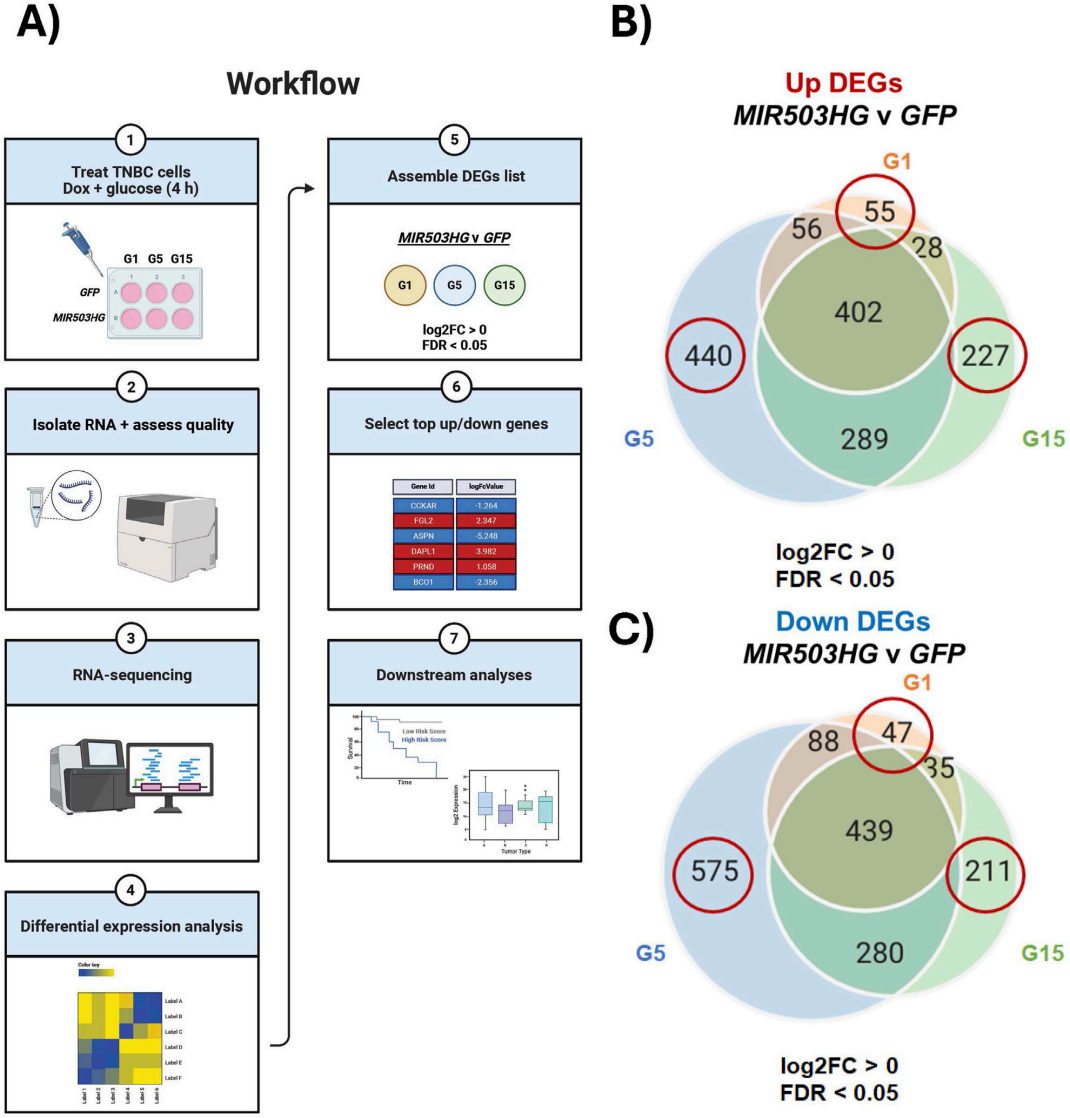
Experimental workflow and RNA-seq expression of differentially expressed genes. The workflow illustrates the experimental design and downstream processes that utilized condition-specific genes. **(A)** Flow chart of experimental design using circled gene sets from Venn diagrams for **(B)** upregulated and **(C)** downregulated genes. Samples include: G1, G5, and G15 for *MIR503HG* and *GFP*. Created with BioRender.com. G1, G5, G15: 1, 5, or 15 g/L of glucose. GFP: Green Fluorescent Protein. DEGs: Differentially Expressed Genes; FDR: False Discovery Change; TNBC: Triple Negative Breast Cancer.

To investigate the role of glucose in *MIR503HG*-dependent gene expression, we conducted RNA-sequencing to identify condition-specific gene sets. Dox-inducible MDA-MB-157 cells were generated to overexpress *MIR503HG* or *GFP*. We resorted to ectopically expressing *MIR503HG* (mature and processed RNA), enabling us to identify *MIR503HG*-regulated gene sets independently of miRNAs. These cells were treated with different glucose concentrations 1 g/L, 5 g/L, and 15 g/L (G1, G5, G15, respectively) for 4 h, while co-treated with doxycycline to induce overexpression of *MIR503HG* or *GFP* (Figure 1A). At 4 h, the cells were collected, and total RNA was isolated (Figure 1A). Differential expression analyses of RNA-sequencing data identified distinct up- and downregulated gene sets for *MIR503HG*-regulated genes compared to *GFP* (Figures 1A,B). The condition-specific differentially expressed genes (DEGs) were utilized to conduct downstream analyses, as shown in Figure 1A, highlighting the biological and clinical significance of glucose on these genes. Figures 1B,C show Venn diagrams for up- and downregulated gene sets, the circled ones of which were used for all subsequent analyses. It was noticed that there were fewer up- and downregulated genes specific to G1, 55 and 47 respectively, compared to the gene sets for G5 and G15 (Figures 1B,C; Supplementary Tables S1–S3).

### Specific Glucose Dose Affects a Distinct Set of *MIR503HG*-Regulated Genes Predicting Clinical Outcomes Across Various Breast Cancer Subtypes

**FIGURE 2:**
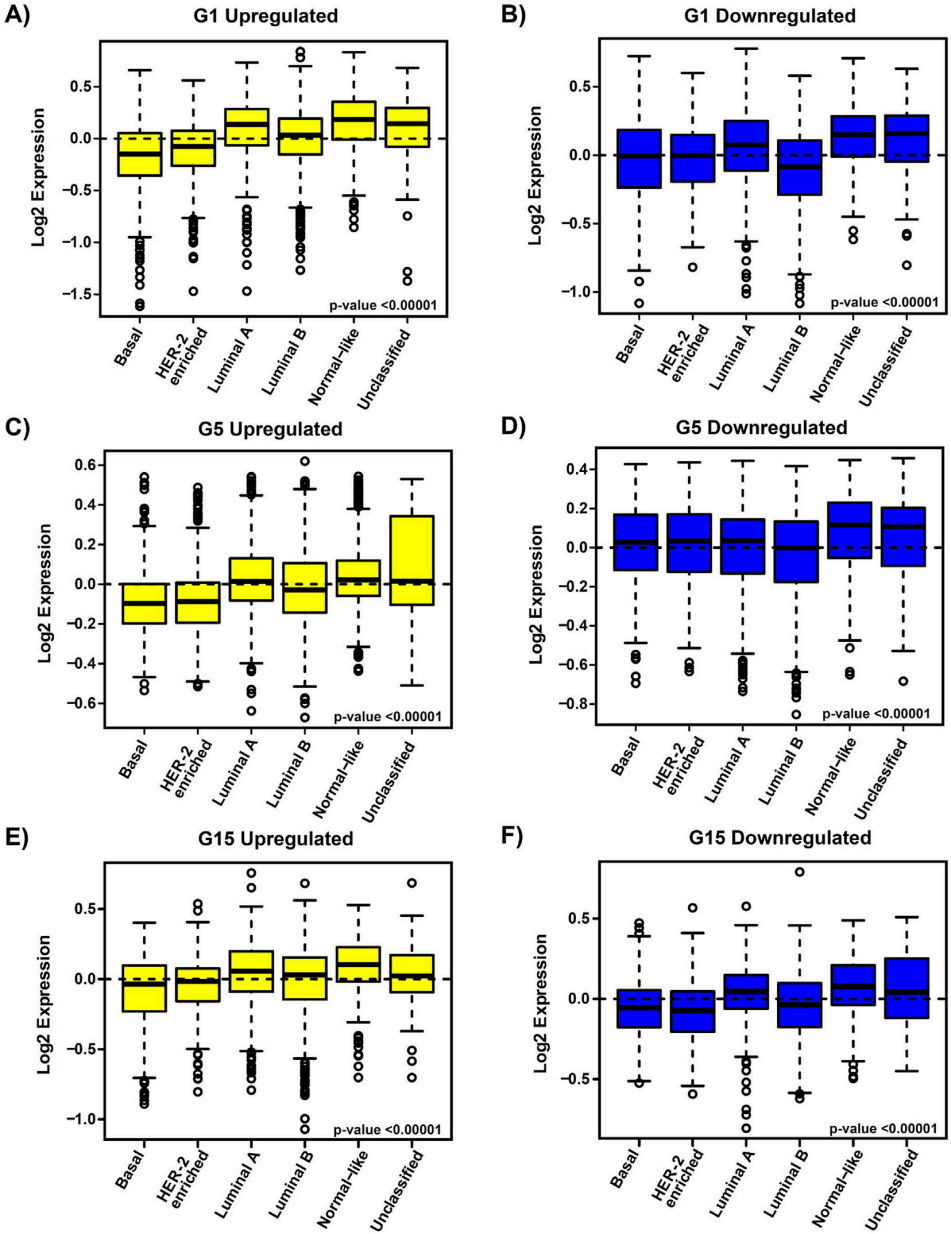
Expression profiles of condition-specific genes’ signature in correlation with breast cancer molecular subtypes in patient tumor samples. Gene signature expression levels according to breast cancer molecular subtype for all tumors, merged gene set, for PAM50 subtypes. Boxplots for **(A)** G1 upregulated, **(B)** G1 downregulated, **(C)** G5 upregulated, **(D)** G5 downregulated, **(E)** G15 upregulated, and **(F)** G15 downregulated gene expression signatures for the following tumor subtypes: Basal (n = 304), HER2-enriched (n = 240), Luminal A (n = 465), Luminal B (n = 471), Normal-like (n = 304) and Unclassified (n = 97). Observed differences are significant by an ANOVA comparison of the means (p-value <0.00001). The dotted line acts as a reference for comparing gene expression levels across different subgroups. G1, G5, G15: 1, 5, or 15 g/L of glucose; HER2: Human Epidermal Growth Factor Receptor 2.

**FIGURE 3:**
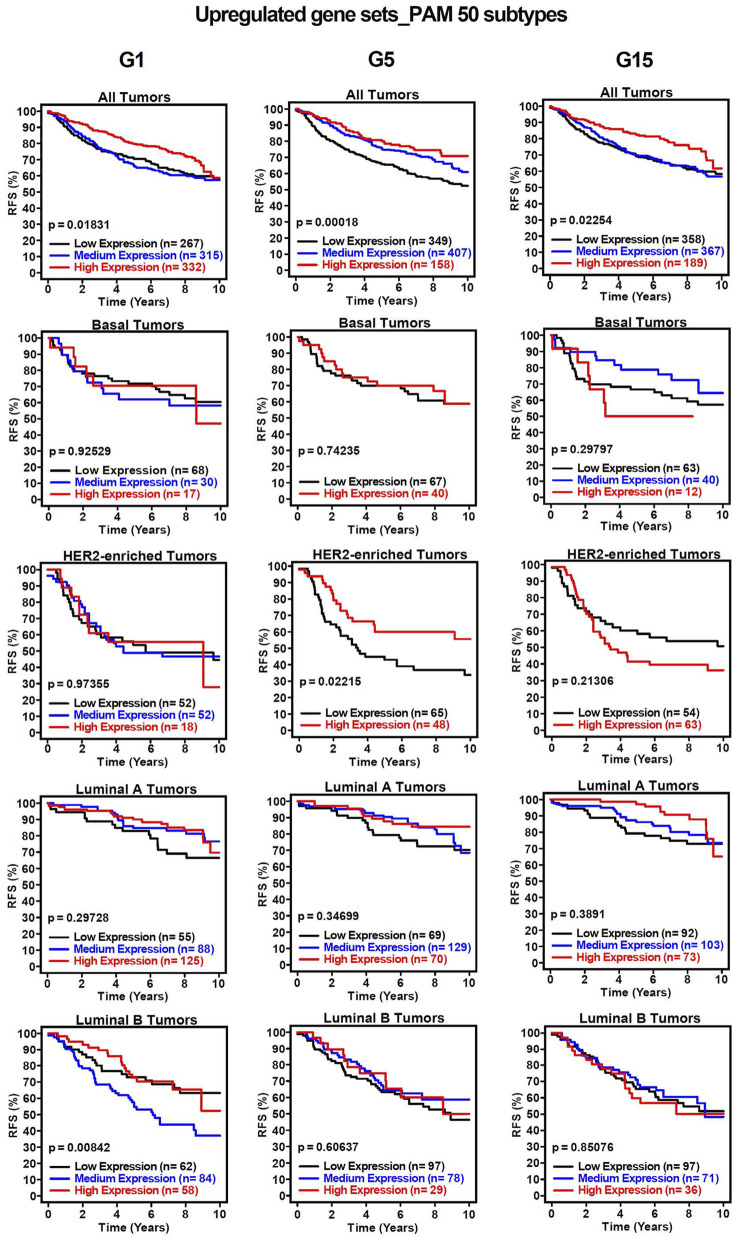
Kaplan-Meier survival analysis for breast cancer subtypes based on upregulated gene signatures shows relapse-free survival rates. Analysis of relapse-free survival rates (RFS) using condition-specific upregulated gene expression signatures. Low expression (black line), medium expression (blue line), and high expression (red line). G1, G5, G15: 1, 5, or 15 g/L of glucose; HER2: Human Epidermal Growth Factor Receptor 2.

**FIGURE 4:**
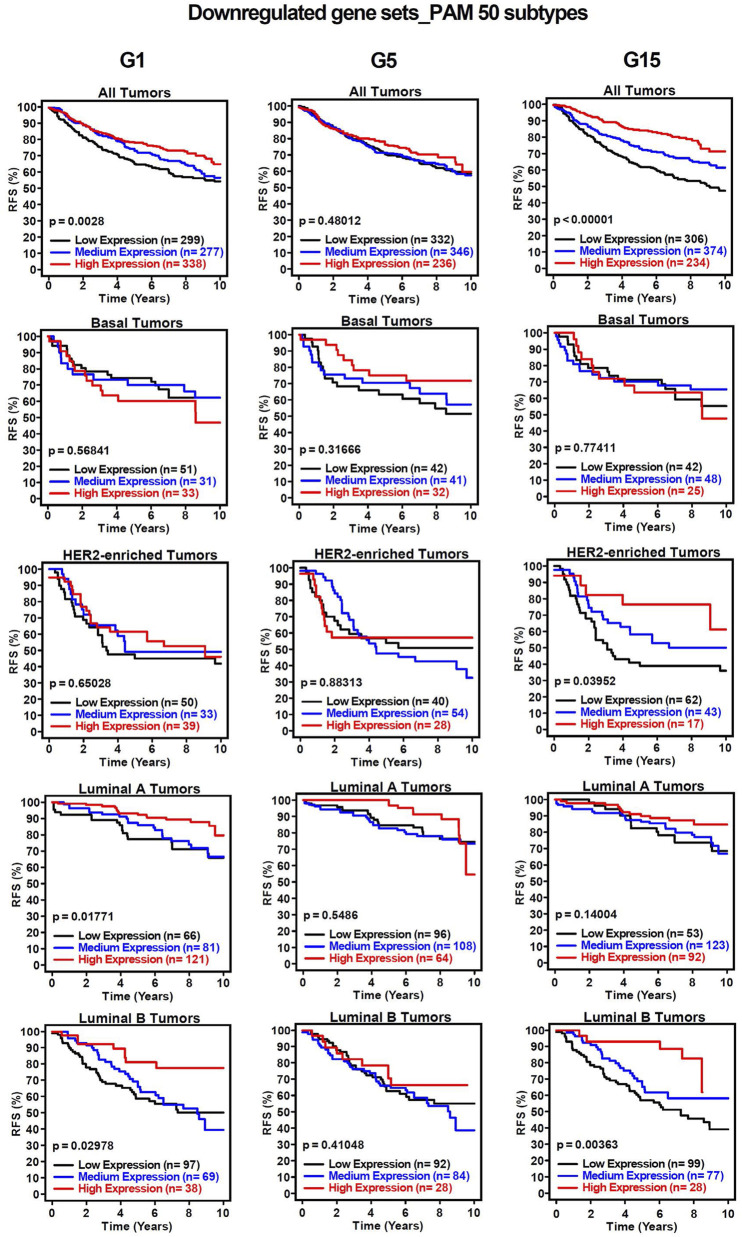
Kaplan-Meier survival analysis for breast cancer subtypes based on downregulated gene signatures shows relapse-free survival rates. Analysis of relapse-free survival rates (RFS) using condition-specific downregulated gene expression signatures. Low expression (black line), medium expression (blue line), and high expression (red line). G1, G5, G15: 1, 5, or 15 g/L of glucose; HER2: Human Epidermal Growth Factor Receptor 2.

To assess the clinical significance of the identified *MIR503HG-*regulated gene sets affected by specific glucose doses, an 1881-sample breast cancer dataset from Gene Expression-Based Outcome for Breast Cancer Online (GOBO) was used. Boxplots were generated to show expression levels of condition-specific gene sets across PAM50 tumors, indicating significant differences among molecular subtypes of breast cancer (Figure 2). Most markedly, the expression levels of G1 and G5 upregulated genes showed attenuation in basal and HER2-enriched tumors, when compared to G15 (Figures 2A,C,E). It was observed that basal tumors displayed similar expression levels to those observed in our condition-specific downregulated *in vitro* analysis (Figures 2B,D,F). Additionally, relapse-free survival (RFS) among molecular breast cancer subtypes was examined using the same condition-specific gene sets used previously. Kaplan-Meier survival analysis indicated varying RFS with increased expression of these genes, however, high expression of these gene sets was significantly associated with better RFS rates in All Tumors for G1, G5 and G15 (Figure 3). Similarly, Kaplan-Meier survival analysis was conducted on condition-specific downregulated gene sets, showing a significant correlation between low gene expression and low RFS rates for G1 and G15, specifically, for All Tumors (Figure 4). Overall survival (OS) and distance metastasis-free survival (DMFS) were also analyzed, however, there was a wider variation of correlation among tumor types and gene set expression levels (Supplementary Figures S1–S4). Together, these findings demonstrate that glucose concentrations exert distinct regulatory effects on *MIR503HG*-regulated genes across breast tumor subtypes, with significant implications for predicting clinical outcomes in breast cancer patients.

### Biological Impact of Glucose-Driven *MIR503HG*-Regulated Genes

**FIGURE 5:**
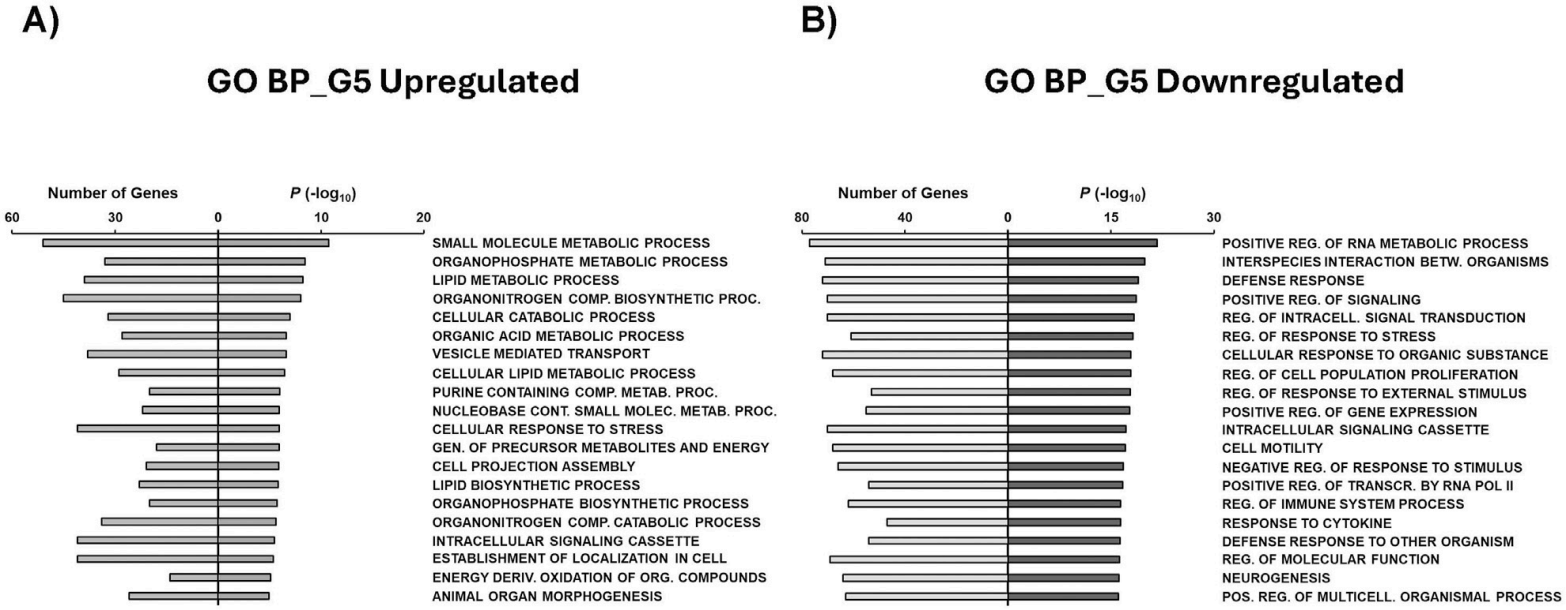
GSEA shows associated functions of condition-specific genes for Biological Processes. Analysis of condition-specific gene sets for biological processes (BP) in cells exposed to G5 condition. **(A)** G5 upregulated and **(B)** G5 downregulated. G5: 5 g/L of glucose; Comp: Compound; Proc: Processes; Metab: Metabolic; Cont: Containing; Molec: Molecule; Gen: Generation; Deriv: Derived; Org: Organic; Reg: Regulation; Betw: Between; Transcr: Transcription; Pos: Positive.

**FIGURE 6:**
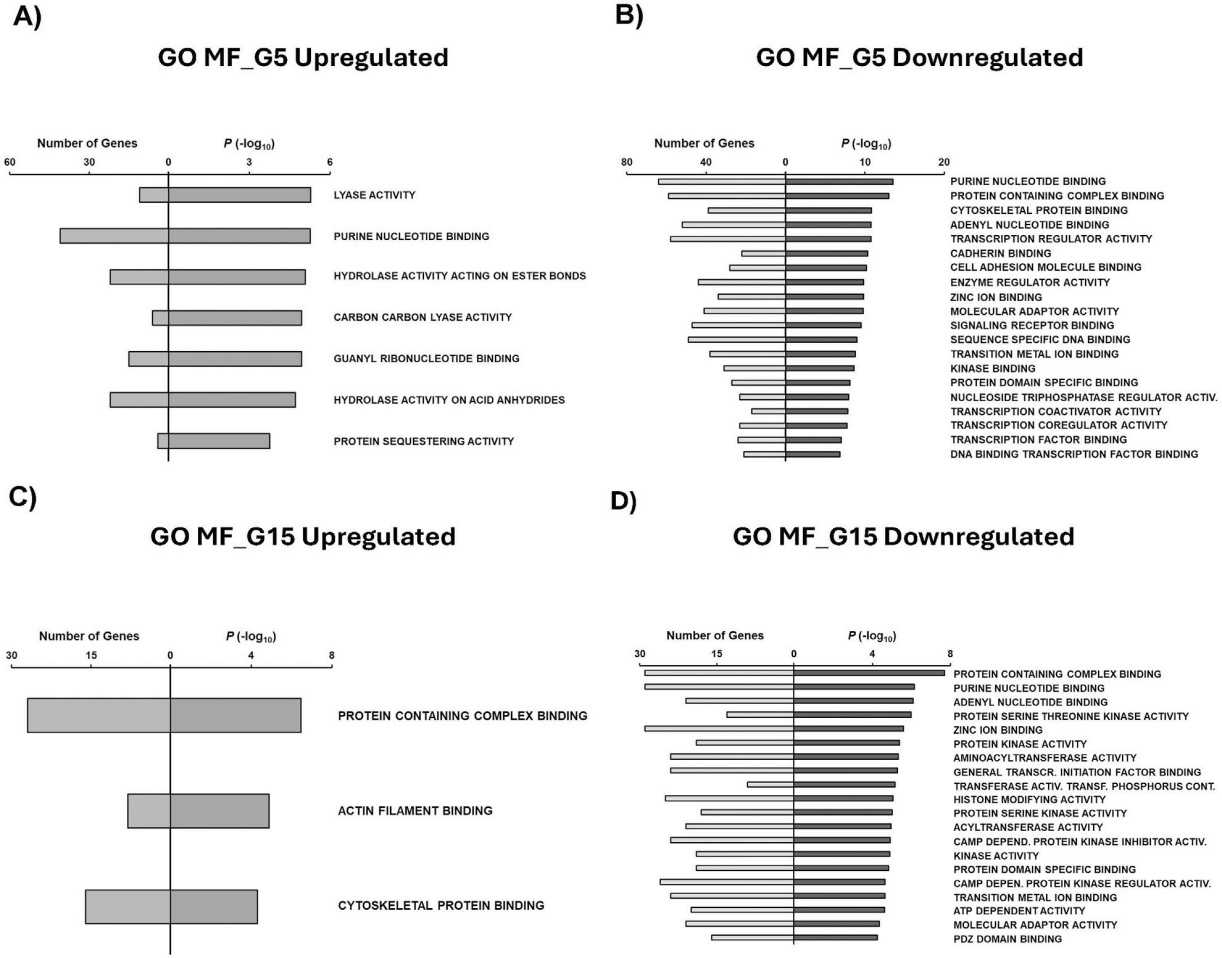
GSEA shows associated functions of condition-specific genes for Molecular Functions. Analysis of condition-specific gene sets for molecular functions (MF) in cells exposed to G5 and G15 conditions. **(A)** G5 upregulated, **(B)** G5 downregulated, **(C)** G15 upregulated, and **(D)** G15 downregulated. G5, G15: 5, or 15 g/L of glucose; Activ: Activity; Transcr: Transcription; Activ: Activity; Transf: Transferring; Depend: Dependent.

**FIGURE 7:**
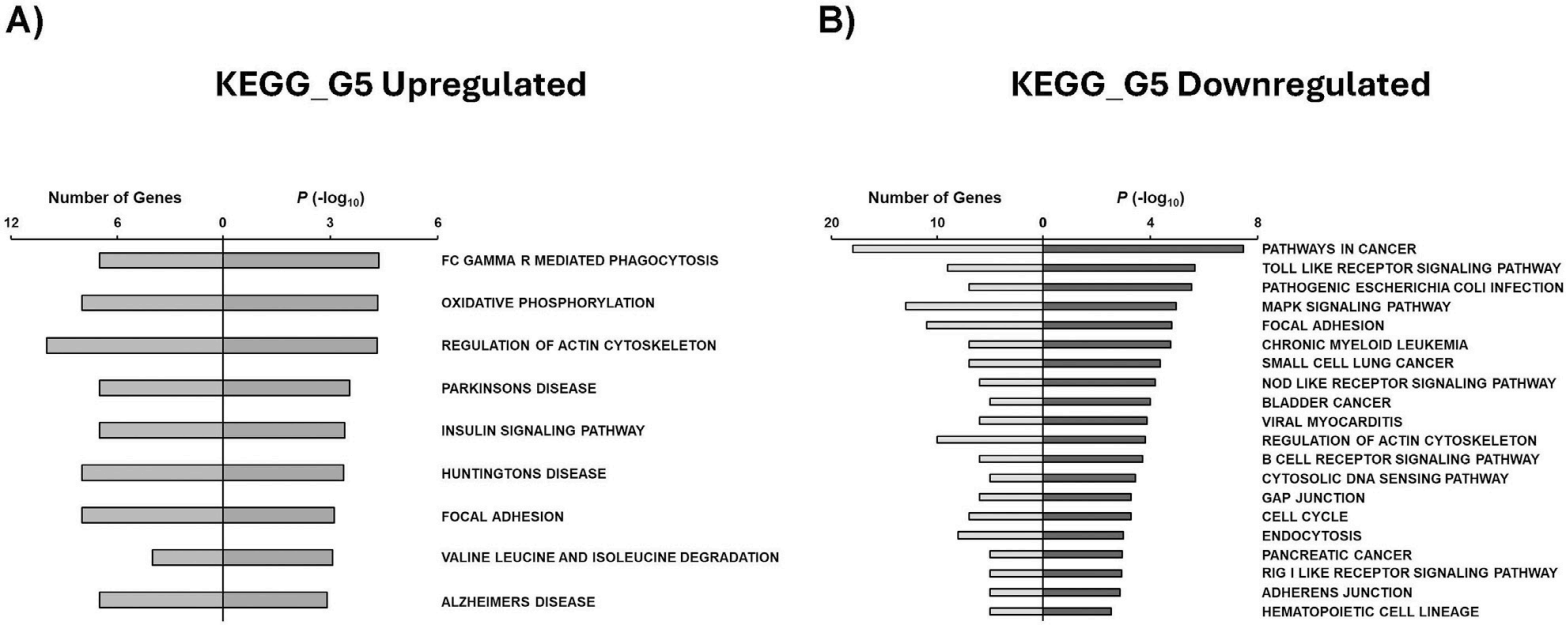
GSEA shows associated functions of condition-specific genes for KEGG pathways. Analysis of condition-specific gene sets for KEGG pathways in cells exposed to G5 condition. **(A)** G5 upregulated and **(B)** G5 downregulated. G5: 5 g/L of glucose; FC: Fragment Crystallizable; R: Receptor.

**FIGURE 8:**
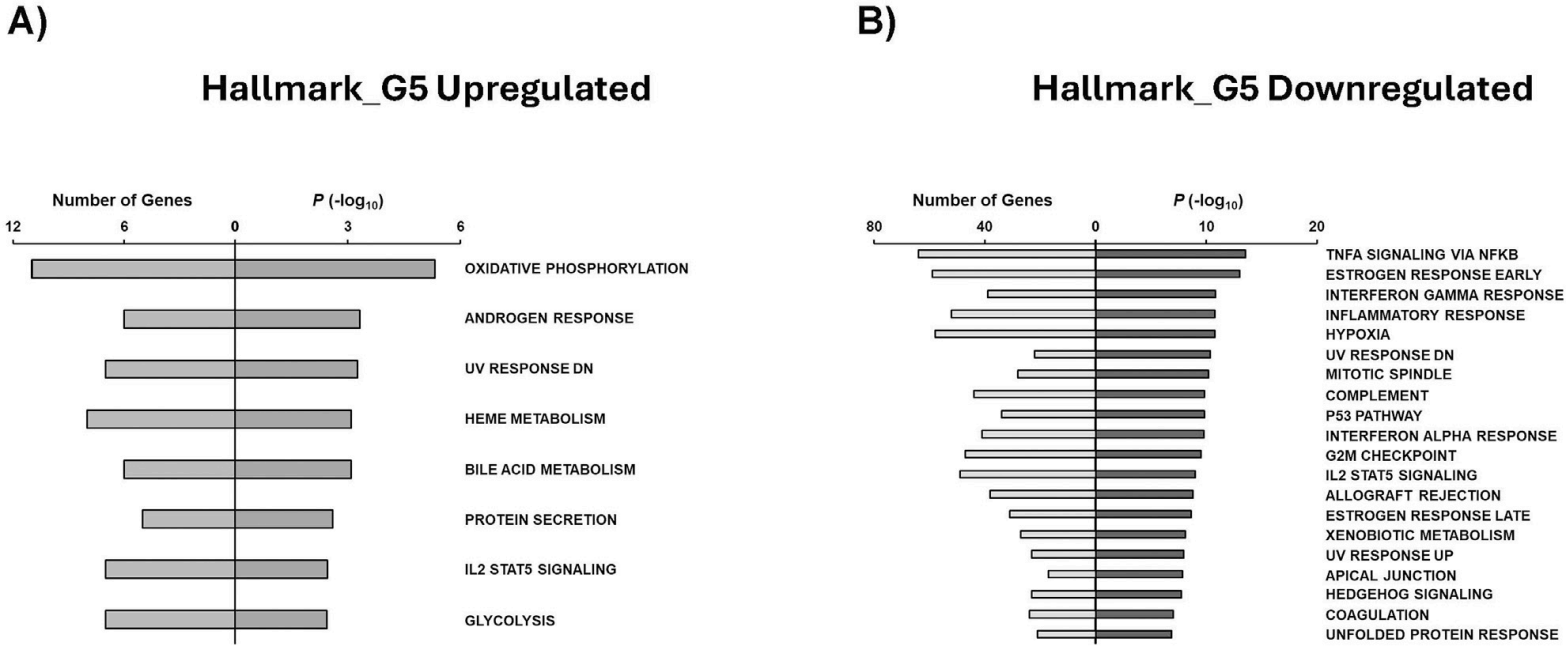
GSEA shows associated functions of condition-specific genes for Hallmark annotations. Analysis of condition-specific gene sets for Hallmark annotations in cells exposed to G5 condition. **(A)** G5 upregulated and **(B)** G5 downregulated. G5: 5 g/L of glucose.

**FIGURE 9:**
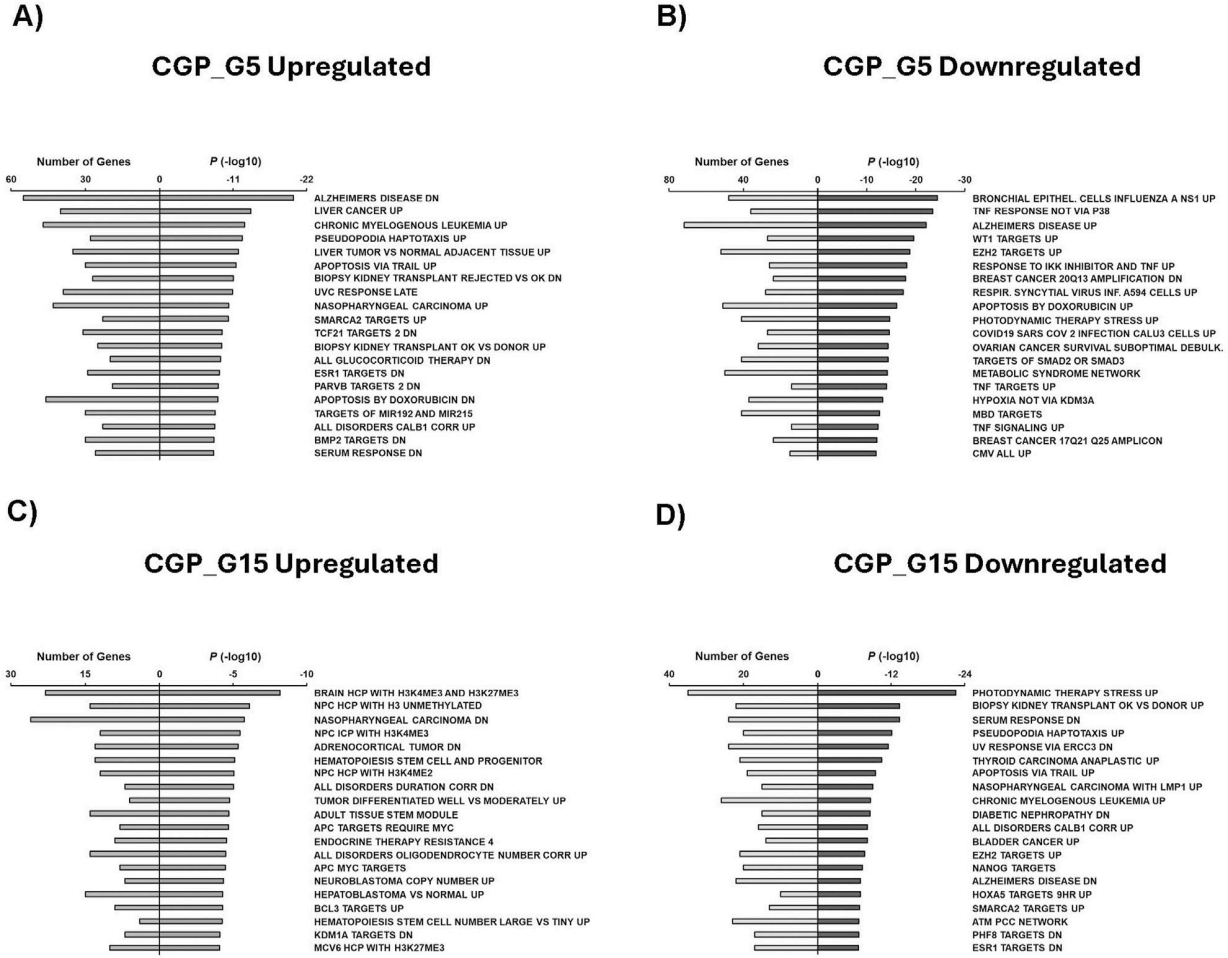
GSEA shows associated functions of condition-specific genes for CGP. Analysis of condition-specific gene sets for chemical and genetic perturbations (CGP) in cells exposed to G5 and G15 conditions. **(A)** G5 upregulated, **(B)** G5 downregulated, **(C)** G15 upregulated, and **(D)** G15 downregulated. G5, G15: 5, or 15 g/L of glucose; DN: Down; VS: Versus; UVC: Ultraviolet light; ESR1: Estrogen Receptor alpha; CORR: Correlation; EPITHEL: Epithelial; TNF: Tumor Necrosis Factor; RESPIR: Respiratory; INF: Infection; DEBULK: Debulking; CORR: Correlation; NCP: Nasopharyngeal Carcinoma; HCP: High CpG-density promoters; ICP: Intermediate-CpG-density promoters; PCC: Pearson’s Correlation Coefficient.

Gene Set Enrichment Analysis (GSEA) was conducted to assess the biological impact of each glucose condition on *MIR503HG*-regulated gene sets. To do this, the previously mentioned condition-specific gene sets were used to derive the top 20 gene ontology (GO) terms, KEGG pathways, Hallmark annotations, and chemical and genetic perturbations (CGP) for each condition, giving insight as to what processes these top up- and downregulated genes are associated with (Figures 5–9; Supplementary Tables S4–S14). Biological processes (BP) for genes upregulated in G5 were mainly involved in metabolic processes relating to small molecules, organophosphates, lipids, cellular catabolic processes, and organic acids (Figure 5A). On the other hand, top BP categories for G5 downregulated genes pertained to regulation of RNA metabolism, signaling, cell proliferation, gene expression, and transcription (Figure 5B). BP for G15 downregulated genes found similar categories to G5 downregulated categories, such as regulation of cell proliferation and signaling, with differences such as proteolysis and phosphorylation observed (Supplementary Figure S5).

Molecular functions (MF) were then obtained to identify molecular roles associated with condition-specific gene sets. Main MF GO terms associated with G5 upregulated genes include lyase activity, purine nucleotide binding, hydrolase activity acting on ester bonds, carbon carbon lyase activity, and guanyl ribonucleotide binding (Figure 6A). Downregulated genes exposed to the G5 condition revealed MF related to purine nucleotide binding, cadherin binding, signaling receptor binding, kinase binding, and transcription factor binding (Figure 6B). Our study found that G15 upregulated genes were primarily associated with MF protein containing complex binding, actin filament binding, and cytoskeletal protein binding (Figure 6C). G15-specific downregulated genes were found to be related to protein-containing complex, protein kinase activity, histone modifying activity, ATP-dependent activity, and PDZ domain binding (Figure 6D).

Similarly, we utilized GSEA to identify KEGG pathways associated with condition-specific gene sets. G5 upregulated genes were observed to be related to Fc gamma R mediated phagocytosis, oxidative phosphorylation, regulation of actin cytoskeleton, insulin signaling pathway, and focal adhesion (Figure 7A). It is also interesting to note that genes related to neurological diseases such as Parkinson’s, Huntington’s, and Alzheimer’s diseases were enriched in the G5 condition. Our study revealed that several KEGG pathways associated with cancer were downregulated in G5, including pathways related to cancer, chronic myeloid leukemia, small cell lung cancer, bladder cancer, and pancreatic cancer (Figure 7B).

Hallmark annotations were also generated for this study. Upregulated genes in G5 were found to be associated mainly with oxidative phosphorylation, androgen response, heme metabolism, bole acid metabolism, and glycolysis (Figure 8A). On the other hand, G5 downregulated Hallmark annotations were related to TNFa signaling via NFKb, estrogen response, inflammatory response, hypoxia, and P53 pathway (Figure 8B). Interestingly, G1 upregulated gene sets were associated with IL2 STAT5 signaling and adipogenesis (Supplementary Figure S6A), while G15 upregulated genes were related to IL6 JAK STAT3 signaling, glycolysis, and myogenesis (Supplementary Figure S6B).

Additionally, our GSEA analysis included investigating chemical and genetic perturbations (CGP). Downregulated genes in G1 condition were mainly associated with ovarian cancer tumors and xenografts down (dn), ELAVL1 targets up, liver cancer ciprofibrate up, liver cancer E2F1 up, liver cancer ACOX1 up, and SATB1 targets dn (Supplementary Figure S7). Top CGP related to G5 upregulated genes include Alzheimer’s disease dn, liver cancer up, chronic myelogenous leukemia up, ESR1 targets up, and apoptosis by doxorubicin dn (Figure 9A). Downregulated G5 genes were primarily associated with bronchial epithelial cells influenza A NS1 up, Alzheimer’s disease up, apoptosis by doxorubicin up, ovarian cancer survival suboptimal debulking, and metabolic syndrome (Figure 9B). CGP related to G15 upregulated genes were mainly associated with brain HCP with H3K4me3 and H3K27me3, nasopharyngeal carcinoma dn, adrenocortical tumor dn, endocrine therapy resistance 4, and neuroblastoma copy number up (Figure 9C). Finally, our study showed that genes related to photodynamic therapy stress up, serum response dn, thyroid carcinoma anaplastic up, EZH2 targets up, and Nanog targets CGP were downregulated in the G15 condition (Figure 9D).

## Discussion

The treatment of TNBC is challenging due to the lack of tumor-specific targets [39]. Chemotherapy, supplemented with surgery, is the typical treatment strategy for TNBC patients [40], though high recurrence rates are still observed after these standard treatment therapies are administered [41]. Several studies have investigated the relationship between glucose and long non-coding RNAs, specifically in breast cancer [42, 43]. The rewiring of metabolic networks in the tumor microenvironment is a highly valuable characteristic among aggressive cancers. It is now known that there are multiple metabolic phenotypes within the TNBC molecular subtype [17], which makes the development of novel treatments a more challenging yet extremely necessary task.

Select lncRNAs, such as *MIR210HG* and *HANR*, have been recognized as modulators of glucose metabolism in TNBC [44, 45]. Additionally, a recent study has explored creating a prognostic risk model based on glucose metabolism-related lncRNAs [43], paving the way for the creation of potential novel therapeutic targets. It is interesting to note that *MIR503HG* has displayed involvement in glucose-related pathologies such as diabetic nephropathy (DN). A study published in 2020 by Cao and Fan showed *MIR503HG* and miR-503-5p expression in human proximal tubular epithelial (HK-2) renal cells was upregulated in high glucose conditions, indirectly promoting apoptosis by suppressing anti-apoptotic protein Bcl-2 [46]. Their findings suggest that this regulatory axis could serve as a potential therapeutic target for diabetic nephropathy. Even though some of the functions of lncRNA *MIR503HG* in TNBC have been identified [31, 47, 48], the transcriptomic effects of glucose on *MIR503HG*-regulated genes are still largely unknown. This prompted us to analyze the associated functions of differentially expressed *MIR503HG*-regulated genes across diverse glucose conditions. One interesting observation in our study is that the condition-specific gene set size for G1 was recognizably smaller than that of G5 and G15, suggesting that there are more shared genes that are differentially regulated in the G1 condition and that glucose affects a very specific *MIR503HG*-regulated gene set at higher concentrations. Through our analysis of breast cancer patient samples using the GOBO database, we observed that the condition-specific genes showed clinical significance in the correlation between gene expression and relapse-free survival. This indicates that these gene sets could be utilized as prognostic markers to predict patient outcomes. Interestingly, genes regulated by *MIR503HG* are associated with less aggressive breast cancer subtypes, such as Luminal A. Except under the G15 condition, expression levels in Basal-like and HER2-enriched tumors were higher for downregulated genes compared to upregulated genes (Figure 2). This could be explained by studies showing that *MIR503HG* functions as a tumor suppressor in ovarian and bladder cancers [30, 49]. Furthermore, this could account for the lack of significant differences in RFS in basal cancer type under different glucose conditions (Figures 3, 4), as the regulated gene sets may be specific to less aggressive cancer subtypes.

During the process of assessing key pathways associated with the identified condition-specific genes, it was noted that many of the regulated genes have neurological functions, as diseases such as Alzheimer’s, Parkinson’s, and Huntington’s were enriched in the G5 condition. It is noteworthy to mention that recent studies have highlighted the fact that basal-type breast cancers disrupt the blood-brain barrier (BBB) during their metastasis to the brain [50], and that glucose transporter 1 (GLUT1) is specifically localized to the BBB [51]. Furthermore, our GSEA analysis of condition-specific gene set revealed associations within the ‘chemical and genetic perturbations’ category, revealed including links to Alzheimer’s disease (G5-upregulated; G5 and G15-downregulated), chromatin modification in brain high-CpG-density promoters (HCPs) (G15-upregulated), and neuroblastoma copy number (G15-upregulated). This correlation requires further in-depth research, as it is an initial investigation of our data.

Furthermore, our study observed key differences in biological function among differentially regulated genes. Genes upregulated in the G5 condition were primarily involved in metabolic processes, while G5-specific downregulated genes were primarily associated with the regulation of gene expression and transcription. This is particularly interesting when compared to the processes related to G15-specific downregulated genes, as the change in dose seemed to not have a profound effect on gene modulation, as processes such as regulation of cell proliferation and signaling were still impacted. More studies are needed, however, as the low gene number between G1-specific and G5-specific groups hindered the ability to conduct further analyses on this comparison.

In conclusion, the differentially expressed *MIR503HG*-regulated genes in various glucose conditions can be utilized to generate diagnostic and prognostic methods for TNBC. Further studies focusing on specific genes will provide additional data for the development of such tools in the future.

## Summary Table

### What Is Known About This Subject

- Triple-negative breast cancer (TNBC) is associated with aggressive tumor phenotypes and higher recurrence rates than other subtypes.
- Recent studies have classified TNBCs into multiple subtypes based on their metabolic phenotype, meaning each subtype must be treated uniquely.
- LncRNA *MIR503HG* has been implicated in TNBC, in which it inhibits cell proliferation.

### What This Paper Adds

- Glucose regulates *MIR503HG*-dependent genes in a dose-dependent manner.
- A distinct set of *MIR503HG*-regulated genes modulated by glucose predicts clinical outcomes across various breast cancer subtypes.
- The crosstalk between glucose and *MIR503HG* in TNBC uncovered novel biological aspects.

This work represents an advance in biomedical science because it reveals that glucose affects genes regulated by noncoding RNAs, specifically *MIR503HG*, suggesting their potential use as therapeutic targets in treating TNBC.

## Data Availability

The datasets presented in this study can be found in online repositories. The names of the repository/repositories and accession number(s) can be found in the article/Supplementary Material.
