# Supplementary material for: Molecular Effects of Glucose on MIR503HG-Regulated Genes in Triple-Negative Breast Cancer
Source: Br J Biomed Sci. 2025 Dec 17;82:15206. doi: 10.3389/bjbs.2025.15206 (PMC12753515; doi:10.3389/bjbs.2025.15206)
Supplement: Supplementary file 6 [file DataSheet1.PDF]

Supplementary Information

"Molecular effects of glucose on *MIR503HG*-regulated genes in triple-negative breast cancer"

Reid *et al.* (2025)

This document contains the following supplementary information: Page

Supplementary Figures 2

• Suppl. Figure 1. Kaplan-Meier survival analysis for breast cancer subtypes based on up-regulated gene signatures shows overall survival.……………………………………………………………2
•Suppl. Figure 2. Kaplan-Meier survival analysis for breast cancer subtypes based on down-regulated gene signatures shows overall survival. ………………………….……………………..3
• Suppl. Figure 3. Kaplan-Meier survival analysis for breast cancer subtypes based on up-regulated gene signatures shows distant metastasis-free survival. ……………………………………...4
• Suppl. Figure 4. Kaplan-Meier survival analysis for breast cancer subtypes based on down-regulated gene signatures shows distant metastasis-free survival. ………………………………..5
• Suppl. Figure 5. GSEA shows associated functions of condition-specific genes for Biological Processes…………………………………………………………………………………………..6
• Suppl. Figure 6. GSEA shows associated functions of condition-specific genes for Hallmark annotations.………………………………………………………………………….............7
• Suppl. Figure 7. GSEA shows associated functions of condition-specific genes for CGP. ………...……………………………………………………………………………….............8

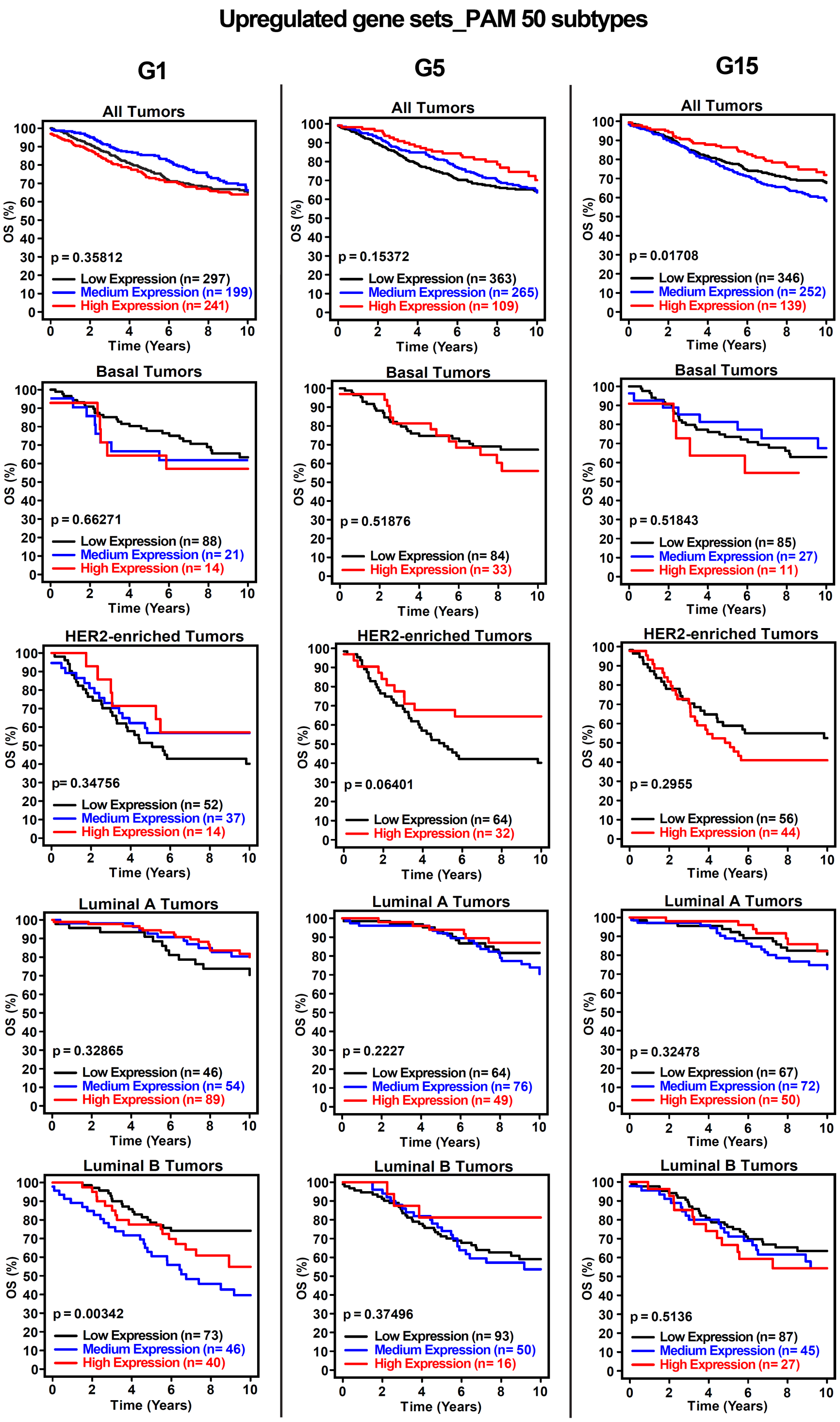

Supplemental Figure 1. Kaplan-Meier survival analysis for breast cancer subtypes based on upregulated gene signatures shows overall survival. Analysis of overall survival (OS) using condition-specific upregulated gene expression signatures. Low expression (black line), medium expression (blue line), and high expression (red line).

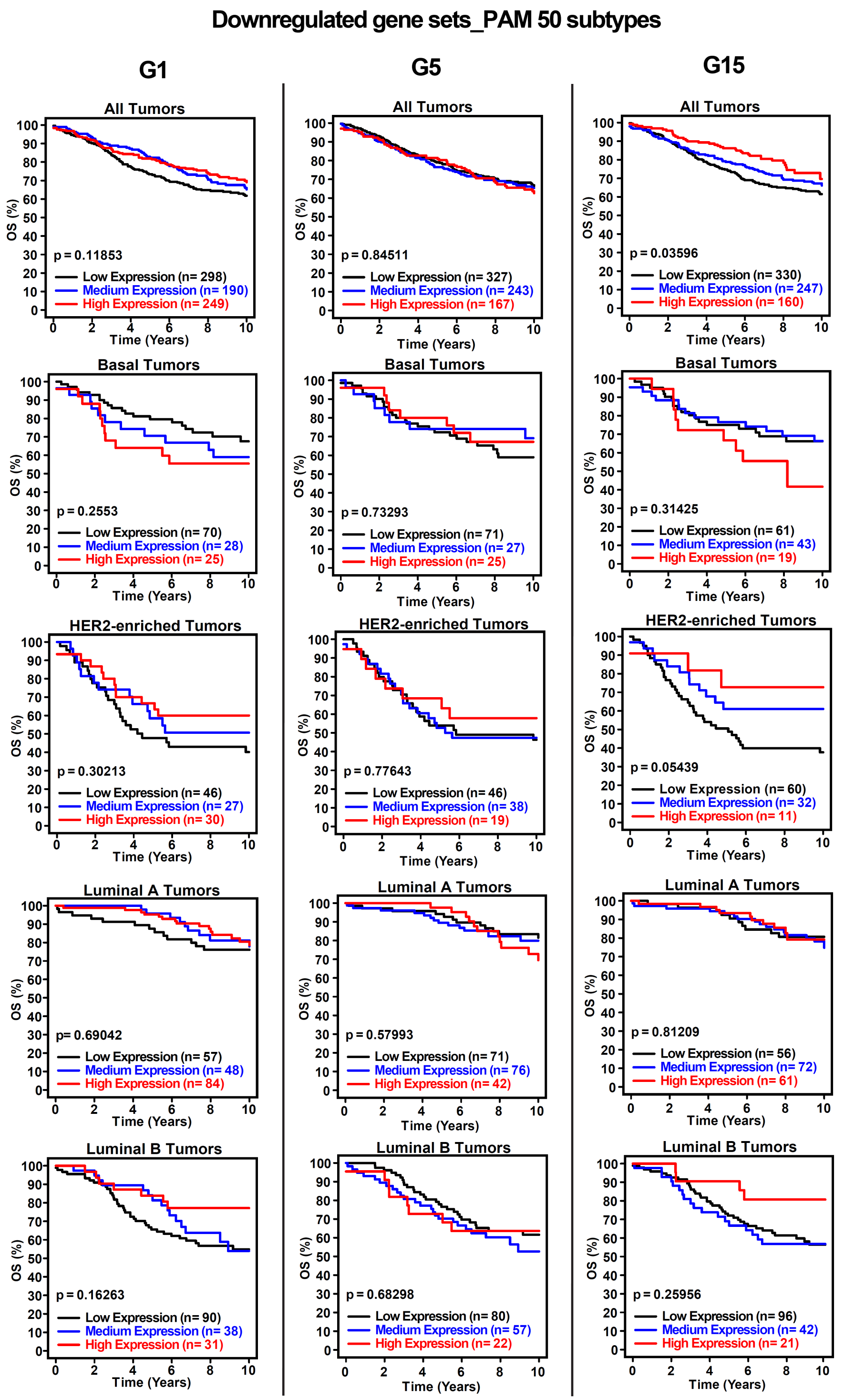

Supplemental Figure 2. Kaplan-Meier survival analysis for breast cancer subtypes based on down-regulated gene signatures shows overall survival. Analysis of overall survival (OS) using condition-specific down-regulated gene expression signatures. Low expression (black line), medium expression (blue line) and high expression (red line).

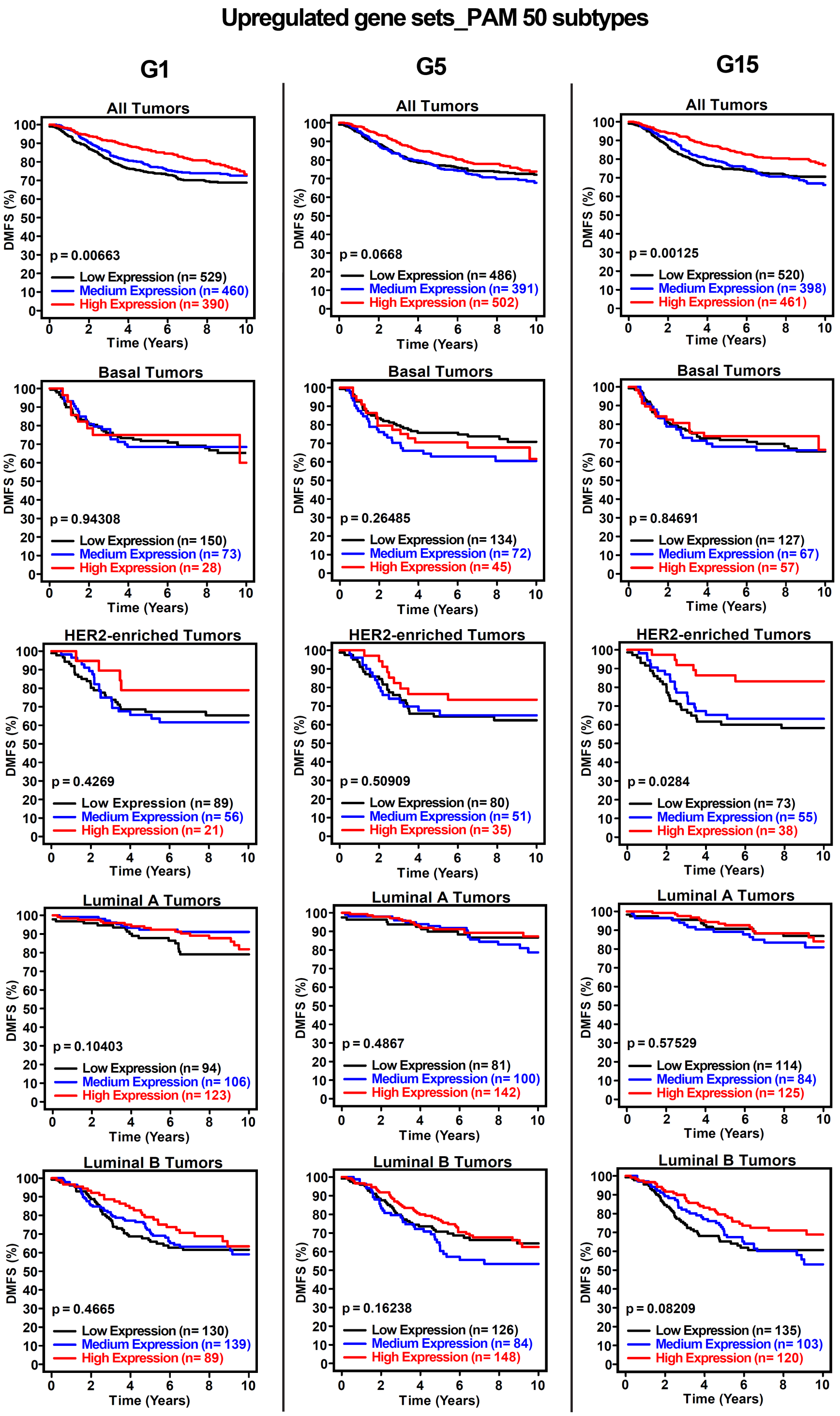

Supplemental Figure 3. Kaplan-Meier survival analysis for breast cancer subtypes based on up-regulated gene signatures shows distant metastasis-free survival. Analysis of distant metastasis-free survival (DMFS) using condition-specific up-regulated gene expression signatures. Low expression (black line), medium expression (blue line) and high expression (red line).

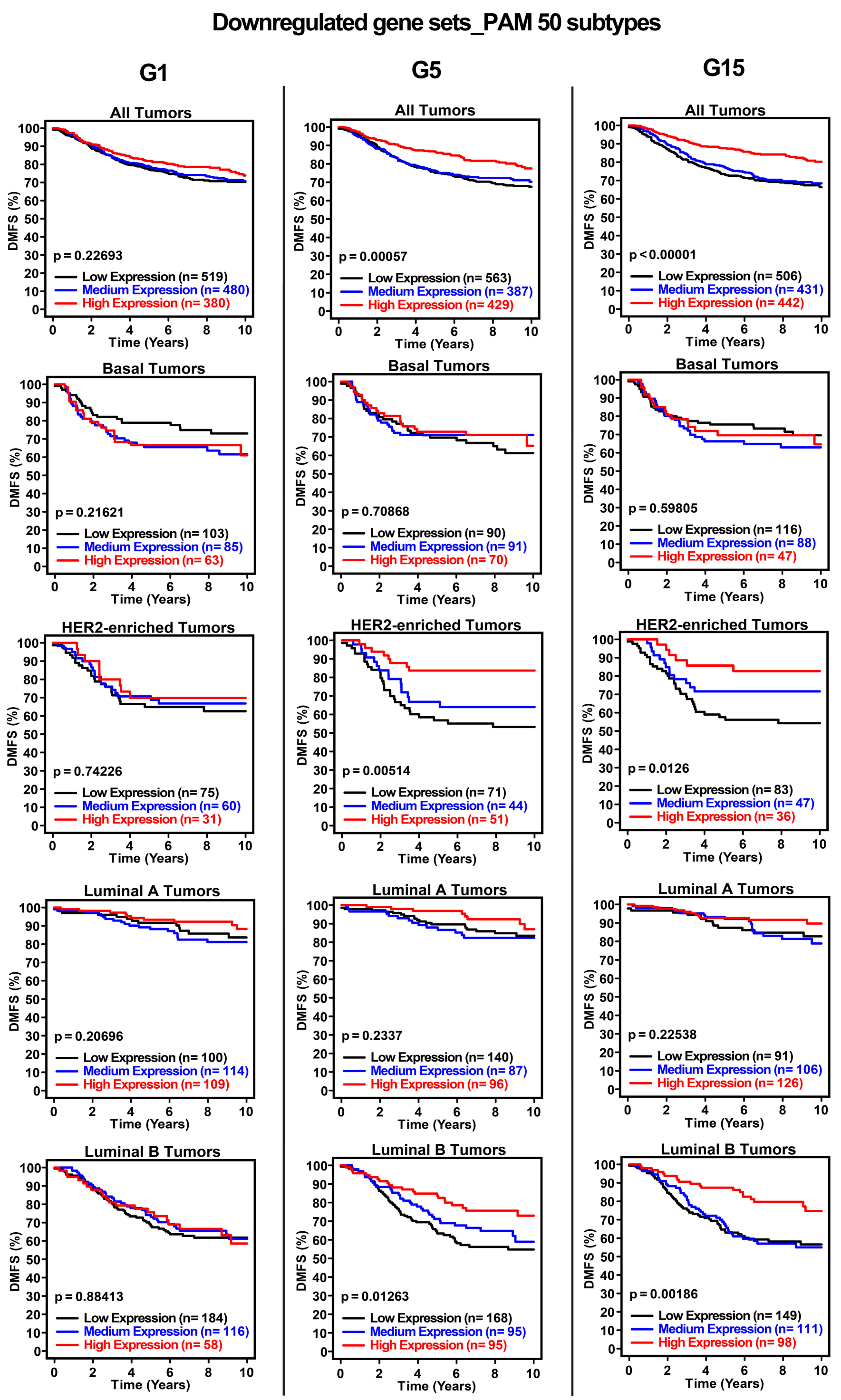

Supplemental Figure 4. Kaplan-Meier survival analysis for breast cancer subtypes based on down-regulated gene signatures shows distant metastasis-free survival. Analysis of distant metastasis-free survival (DMFS) using condition-specific down-regulated gene expression signatures. Low expression (black line), medium expression (blue line), and high expression (red line).

GO BP_G15 Downregulated

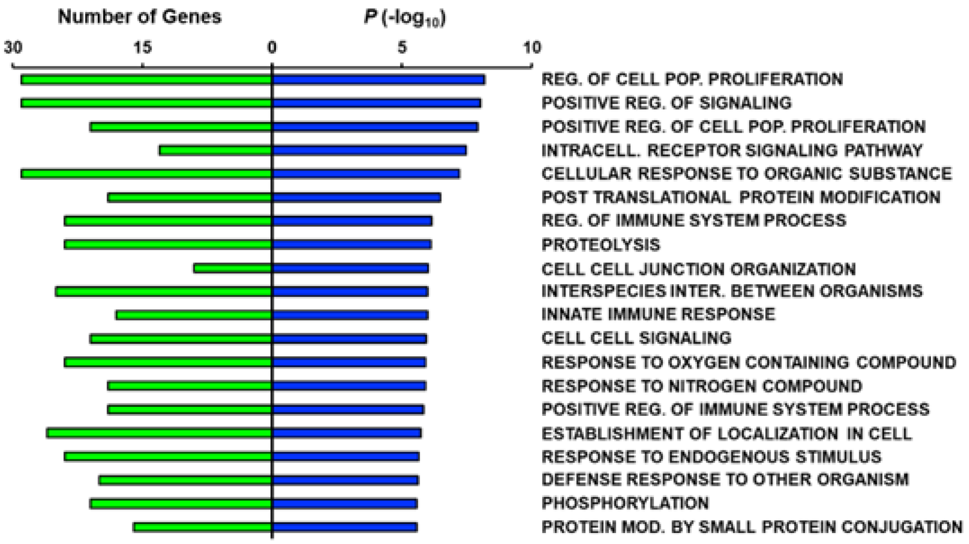

Supplemental Figure 5. GSEA shows associated functions of condition-specific genes for Biological Processes. Analysis of down-regulated condition-specific gene sets for biological processes (BP) in cells exposed to G15 condition.

A)

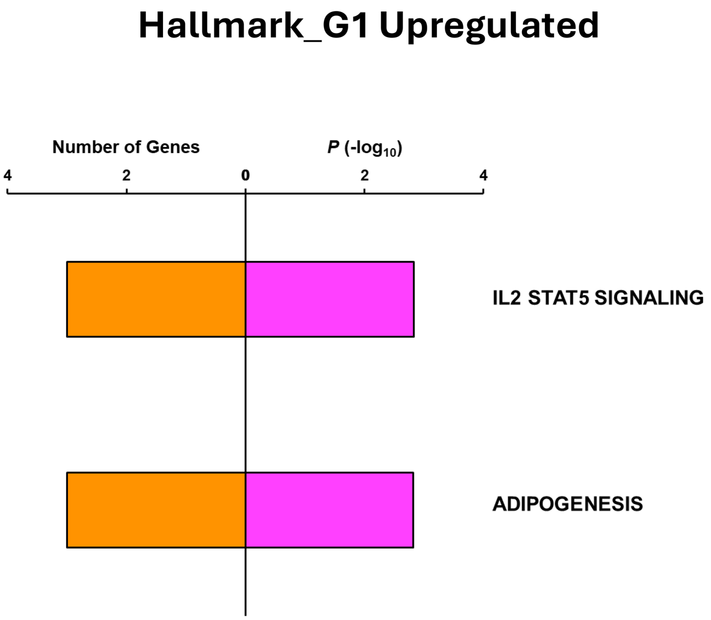

B)

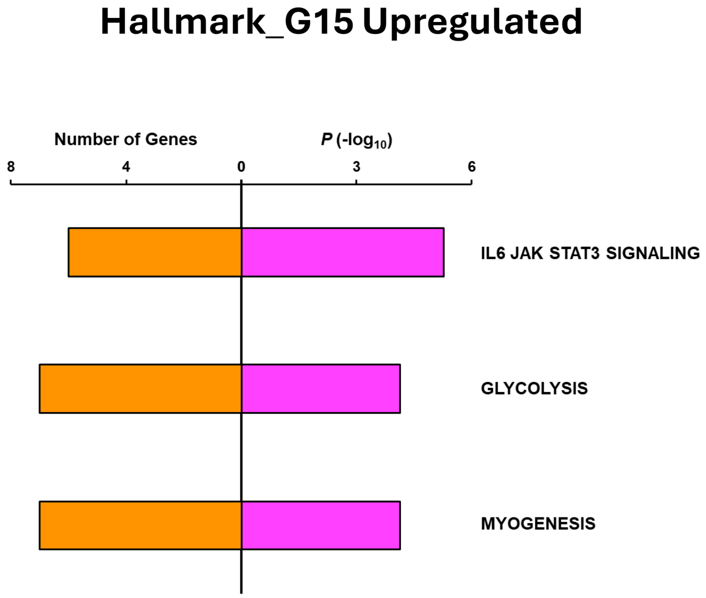

Supplemental Figure 6. GSEA shows associated functions of condition-specific genes for Hallmark annotations. Analysis of up-regulated condition-specific gene sets for Hallmark annotations in cells exposed to **A)** G1 condition and **B)** G15 condition.

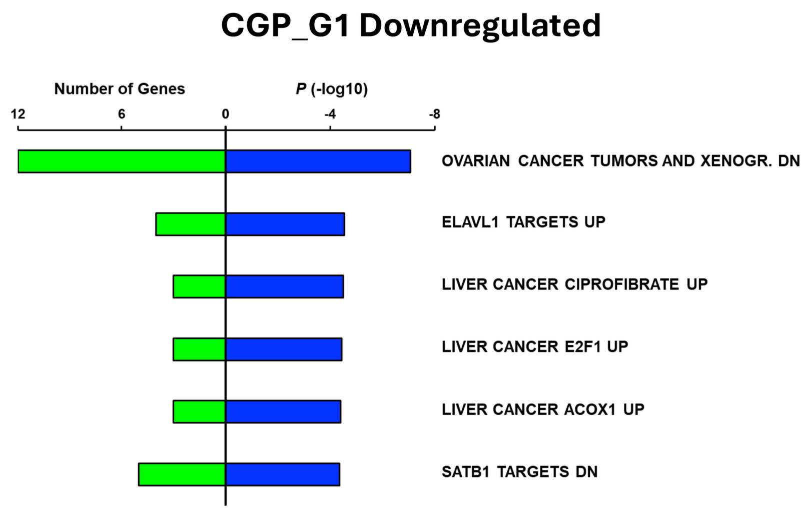

Supplemental Figure 7. GSEA shows associated functions of condition-specific genes for CGP. Analysis of down-regulated condition-specific gene sets for chemical and genetic perturbations (CGP) in cells exposed to G1 condition. Down-regulated (DN) and up-regulated (UP), respectively.
